# Comparative Diagnostic Accuracy of Clinical Assessment, Computed Tomography (CT), and Magnetic Resonance Imaging With Magnetic Resonance Cholangiopancreatography (MRI/MRCP) in Evaluating Common Bile Duct Stones

**DOI:** 10.7759/cureus.94572

**Published:** 2025-10-14

**Authors:** Mina Al-Dulaimi, Mustafa Ibraheem, Mustafa Abdulkareem, Aaisha Al-Dujaili, Ameer Abdulkareem

**Affiliations:** 1 Stroke, Shrewsbury and Telford Hospital NHS Trust, Telford, GBR; 2 Internal Medicine, Shrewsbury and Telford Hospital NHS Trust, Telford, GBR; 3 Emergency Medicine, Shrewsbury and Telford Hospital NHS Trust, Telford, GBR; 4 Family Medicine, Shaikh Zayed Hospital, Baghdad, IRQ; 5 Trauma and Orthopedics, Shaikh Zayed Hospital, Baghdad, IRQ

**Keywords:** clinical assessment, common bile duct stone, comparative analysis, ct, mri/mrcp

## Abstract

Purpose: To determine the most effective method for diagnosing common bile duct (CBD) stones, this research retrospectively compares the diagnostic accuracy of initial clinical evaluation, computed tomography (CT), and magnetic resonance imaging with magnetic resonance cholangiopancreatography (MRI/MRCP). The goal is to identify the optimal non-invasive strategy to guide patient management and prevent unnecessary invasive procedures such as endoscopic retrograde cholangiopancreatography (ERCP). An accurate diagnosis of choledocholithiasis is essential for proper patient care.

Materials and methods: This single-center, retrospective analysis involved 691 adult patients who were under evaluation for suspected choledocholithiasis. The effectiveness of clinical assessment, multidetector CT, and MRI/MRCP was measured against definitive diagnoses obtained from surgical or procedural findings. For each diagnostic method, the study calculated sensitivity, specificity, accuracy, and the area under the receiver operating characteristic curve (AUC).

Results: CBD stones were present in 311 of the 691 patients, a prevalence of 45%. MRI/MRCP was the most effective diagnostic tool, with a sensitivity of 92.7%, a specificity of 91.9%, and an accuracy of 92.3%. In comparison, CT scans yielded a sensitivity of 73.2%, a specificity of 82.9%, and an accuracy of 78.3%. Clinical assessment resulted in a sensitivity of 71.1%, a specificity of 87.1%, and an accuracy of 80%. The AUC for MRI/MRCP (0.95) was markedly higher than that for both CT (0.83; p<0.001) and clinical assessment (0.81; p<0.001). There was no significant difference in the diagnostic performance between CT and clinical assessment (p=0.45).

Conclusion: For the non-invasive identification of CBD stones, MRI/MRCP provides superior diagnostic accuracy compared to both CT and clinical evaluation. It is recommended as the top imaging choice for patients with suspected choledocholithiasis.

## Introduction

Choledocholithiasis, the presence of stones in the common bile duct (CBD), is a significant clinical problem, affecting 3-16% of patients with gallstones [1]. If left untreated, CBD stones can lead to serious complications, including biliary obstruction, acute cholangitis, pancreatitis, and secondary biliary cirrhosis [2]. The timely and accurate diagnosis of choledocholithiasis is therefore crucial for guiding appropriate management, which often involves therapeutic endoscopic retrograde cholangiopancreatography (ERCP) [3].

The diagnostic pathway for suspected CBD stones typically begins with clinical assessment and laboratory testing. Guidelines from professional bodies, such as the American Society for Gastrointestinal Endoscopy (ASGE), use a combination of clinical predictors and liver function tests to stratify patients into low-, intermediate-, or high-risk categories for choledocholithiasis [4]. While this clinical assessment is vital for triage, its diagnostic accuracy alone is limited. Consequently, imaging plays a central role. Transabdominal ultrasound (US) is the standard first-line imaging modality due to its non-invasiveness and wide availability. However, its sensitivity for directly visualizing CBD stones is highly variable and often suboptimal, particularly in the presence of overlying bowel gas or patient obesity [5].

When the initial evaluation is inconclusive, more advanced imaging is required. Computed tomography (CT) is frequently performed in the emergency setting due to its speed and ability to evaluate for alternative causes of abdominal pain. However, conventional CT has recognized limitations in detecting CBD stones, especially those that are small or isodense to bile [6,7]. Magnetic resonance imaging with magnetic resonance cholangiopancreatography (MRI/MRCP) has emerged as a highly accurate, non-invasive alternative. By using heavily T2-weighted sequences, MRCP can exquisitely delineate the biliary tree and identify intraductal filling defects without the need for ionizing radiation or contrast agents [8].

While the general superiority of MRI/MRCP is widely accepted, there remains a need for a robust study that directly compares the diagnostic performance of clinical assessment strategies, multidetector CT, and high-field MRI/MRCP within a single, large patient cohort. Quantifying the precise diagnostic accuracy of each step in the modern diagnostic algorithm is essential for optimizing patient care and resource utilization. Therefore, the aim of our study was to retrospectively compare the diagnostic accuracy of clinical assessment, CT, and MRI/MRCP against a surgical or endoscopic reference standard in the evaluation of CBD stones, thereby defining the optimal non-invasive pathway for patient selection for ERCP.

## Materials and methods

Study design and ethical approval

This was a retrospective, single-hospital comparative diagnostic accuracy study conducted at Martyr Al-Sadr General Hospital in Baghdad, Iraq. Institutional review board approval was waived at Al-Rusafa Health Center to access the hospital's electronic medical record (EMR).

Patient population

**Figure 1:**
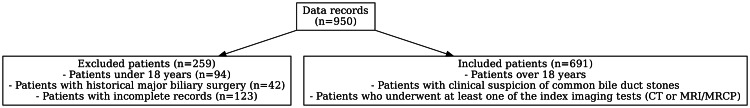
Flowchart of patient selection CT: computed tomography; MRI/MRCP: magnetic resonance imaging with magnetic resonance cholangiopancreatography

A computerized search of the hospital's EMR was performed to identify adult patients who underwent evaluation for suspected choledocholithiasis. A total of 950 patient records were initially screened for eligibility between January 2023 and January 2025. After applying inclusion and exclusion criteria, a final cohort of 691 (72.7%) patients was included in the analysis (Figure 1).

Data collection

Data for all 691 patients were retrospectively extracted from the EMR. A standardized data collection form was used to capture demographics, clinical presentation (symptoms and signs), and laboratory values. The reference standard for the final diagnosis was established by one or more of the following: (1) direct visualization and successful extraction of stones during ERCP, (2) visualization of stones on intraoperative cholangiography (IOC), or (3) direct surgical exploration of the CBD with stone extraction. The time interval between the index imaging test (CT or MRI/MRCP) and the definitive reference procedure was required to be within two weeks to minimize bias from stone passage or formation.

Clinical assessment criteria

The clinical assessment was based on the ASGE high-risk criteria for choledocholithiasis. A patient was categorized as "positive" on clinical assessment if they met one of the following "very strong" predictors: (1) presence of a CBD stone on a prior transabdominal US, (2) clinical presentation of acute cholangitis (Charcot's triad or Reynolds' pentad), or (3) a total serum bilirubin >4 mg/dL. A patient was also considered high-risk if they presented with a combination of two "strong" predictors: a dilated CBD (>6 mm) on US and a total serum bilirubin between 1.8 and 4 mg/dL.

Imaging protocols

**Figure 2:**
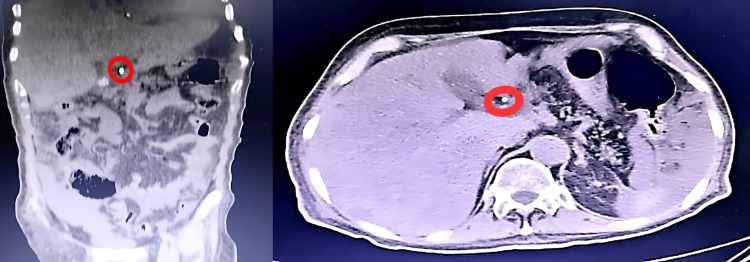
High-resolution axial and coronal contrast-enhanced CT images of the abdomen in a 62-year-old man with jaundice. (a) Coronal CT image shows a hyperdense (calcified) focus within the distal common bile duct (red circle). (b) Axial CT image confirms the hyperdense intraluminal focus, consistent with choledocholithiasis. Note the mild dilation of the upstream biliary duct CT: computed tomography

CT examinations were performed on 64- or 128-detector row scanners. The protocol included a non-contrast acquisition of the upper abdomen followed by a portal venous phase acquisition 70 seconds after injection of a non-ionic, iodinated contrast agent (3-4 mL/s). Images were reconstructed in 3 mm axial, coronal, and sagittal planes (Figure 2).

**Figure 3:**
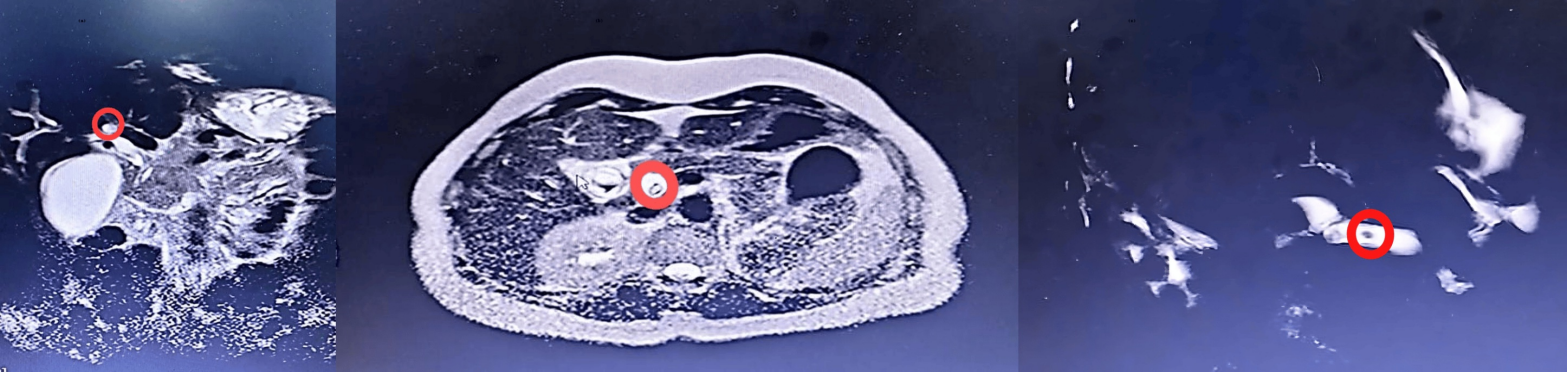
Coronal and axial T2-weighted MRCP images in a 55-year-old woman with right upper quadrant pain. (a) Axial MRCP image demonstrates a low-signal (dark) oval filling defect within the distal common bile duct (red circle). (b) Coronal MRCP thick-slab image clearly delineates the same low-signal filling defect (red circle) against the bright fluid-filled biliary tree. (c) Additional oblique slab confirms the presence of the common bile duct stone with surrounding high-signal bile MRCP: magnetic resonance cholangiopancreatography

MRI examinations were performed on 1.5T or 3T scanners using a phased-array body coil. The protocol included axial and coronal T2-weighted half-Fourier acquisition single-shot turbo spin-echo (HASTE)/single-shot fast spin echo (SS-FSE) sequences, pre-contrast T1-weighted sequences, diffusion-weighted imaging (DWI), and a high-resolution, heavily T2-weighted 3D MRCP sequence for the detailed visualization of the biliary tree (Figure 3).   

It should be noted that variations in scanner vendor and specific sequence parameters across the participating hospitals were present, limiting exact procedural reproducibility.

Image analysis

All CT and MRI/MRCP examinations were retrospectively reviewed by two board-certified abdominal radiologists (>10 years' experience), who were blinded to the final diagnosis and other test results. Discrepancies were resolved by a third senior radiologist (over 20 years' experience). A positive finding for choledocholithiasis on CT was defined as an unequivocal filling defect within the CBD. This blinding process was implemented to minimize interpretation bias. A positive finding on MRI/MRCP was defined as a persistent, low-signal-intensity filling defect within the bile on heavily T2-weighted sequences. A formal analysis of interobserver agreement (e.g., calculation of kappa statistics) was not performed as part of this study.

Statistical analysis

Data analysis was carried out using IBM SPSS Statistics for Windows, Version 28.0 (IBM Corp., Armonk, New York, United States). Foundational characteristics of the study population were outlined using descriptive statistics, expressed as mean±standard deviation and counts with percentages. The performance of each diagnostic test was evaluated based on the following classifications: true positive (TP): the test correctly indicated the presence of CBD stones, which was confirmed by the final diagnosis; false positive (FP): the test incorrectly indicated the presence of CBD stones when they were absent; true negative (TN): the test correctly indicated the absence of CBD stones, aligning with the final diagnosis; and false negative (FN): the test incorrectly indicated the absence of CBD stones when they were present.

Based on these categories, several metrics for diagnostic performance were determined, along with their 95% confidence intervals (CIs): sensitivity: a measure of a test's capacity to accurately detect patients who have the disease and calculated as $\frac{TP}{TP + FN}$; specificity: a measure of a test's capacity to accurately identify patients who do not have the disease and calculated as $\frac{TN}{TN + FP}$; positive predictive value (PPV): the likelihood that a patient with a positive test result is actually afflicted with the disease and calculated as $\frac{TP}{TP + FP}$; negative predictive value (NPV): the likelihood that a patient with a negative test result is truly free of the disease and calculated as $\frac{TN}{TN + FN}$; accuracy: the overall proportion of tests that provided a correct result and calculated as $\frac{TP+TN}{TP+FP+TN+FN}​​​​​​​$; and diagnostic odds ratio (DOR): a single metric that encapsulates test performance, where a higher value indicates better discriminatory ability, and calculated as $\frac{TP*TN}{FP*FN}​​​​​​​$​​​​​​​​​​​​​.

To compare the sensitivity and specificity between paired diagnostic methods, the McNemar test was utilized. Furthermore, a receiver operating characteristic (ROC) curve analysis was performed, and the DeLong test was used to compare the area under the curve (AUC). A p-value below 0.05 was considered to indicate statistical significance.

## Results

A total of 691 patients were included in the final analysis. The mean age of the cohort was 58.7±15.2 years, and 362 (52.4%) patients were female. Right upper quadrant tenderness was the most common clinical sign, present in 473 (68.5%) patients. The mean peak total bilirubin was 5.2±2.8 mg/dL. Baseline demographic, clinical, and laboratory characteristics are summarized in Table 1.

**Table 1:** Baseline demographic, clinical, and laboratory characteristics of the study population (n=691)

Variable		Value (n (%)/mean±SD)
Patient demographics		
Age (years)		58.7±15.2
Gender	Male	329 (47.6%)
	Female	362 (52.4%)
Clinical presentation		
Symptoms	Jaundice	329 (47.6%)
	Right upper quadrant pain	405 (58.6%)
	Vomiting	149 (21.6%)
	Fever	107 (15.5%)
Symptom duration (hours)		43.5
Signs	Right upper quadrant tenderness	473 (68.5%)
	Scleral icterus/skin jaundice	378 (54.7%)
	Murphy's sign	158 (22.9%)
Laboratory values		
Total bilirubin (mg/dL)		5.2±2.8
Direct bilirubin (mg/dL)		4.8±1.2
Alkaline phosphatase (ALP) (U/L)		380±210
Aspartate aminotransferase (AST) (U/L)		90.5±25.41
Alanine aminotransferase (ALT) (U/L)		105±31.56
White blood cell (WBC) count (×10⁹/L)		10±4.5
Amylase/lipase (U/L)		134.95±50.61

The final diagnosis confirmed the presence of CBD stones in 311 (45%) patients of the cohort. MRI/MRCP demonstrated superior diagnostic performance for CBD stone detection with a sensitivity of 92.7% (95% CI: 87.7-96.1), a specificity of 91.9% (95% CI: 87.0-95.4), and an overall accuracy of 92.3% (95% CI: 88.9-94.8). In comparison, CT scans showed a sensitivity of 73.2% (95% CI: 66.7-79.0), a specificity of 82.9% (95% CI: 77.4-87.4), and an accuracy of 78.3% (95% CI: 74.1-82.1). Clinical assessment yielded a sensitivity of 71.1% (95% CI: 65.8-75.9), a specificity of 87.1% (95% CI: 83.5-90.1), and an accuracy of 80% (95% CI: 76.9-82.9). Detailed performance metrics are provided in Table 2.

**Table 2:** Diagnostic performance of clinical assessment, CT, and MRI/MRCP for CBD stone detection TP: true positive; FP: false positive; TN: true negative; FN: false negative; PPV: positive predictive value; NPV: negative predictive value; DOR: diagnostic odds ratio; CT: computed tomography; MRI/MRCP: magnetic resonance imaging with magnetic resonance cholangiopancreatography; CBD: common bile duct

Modality	Clinical assessment	CT	MRI/MRCP
N	691	420	350
TP	221	145	153
FP	49	38	15
TN	331	184	170
FN	90	53	12
Sensitivity % (95% CI)	71.1 (65.8-75.9)	73.2 (66.7-79.0)	92.7 (87.7-96.1)
Specificity % (95% CI)	87.1 (83.5-90.1)	82.9 (77.4-87.4)	91.9 (87.0-95.4)
PPV % (95% CI)	81.9 (76.9-86.0)	79.2 (72.8-84.7)	91.1 (86.0-94.7)
NPV % (95% CI)	78.6 (74.6-82.2)	77.6 (72.2-82.4)	93.4 (88.8-96.5)
DOR % (95% CI)	16.6	13.3	144.5
Accuracy % (95% CI)	80.0 (76.9-82.9)	78.3 (74.1-82.1)	92.3 (88.9-94.8)

**Figure 4:**
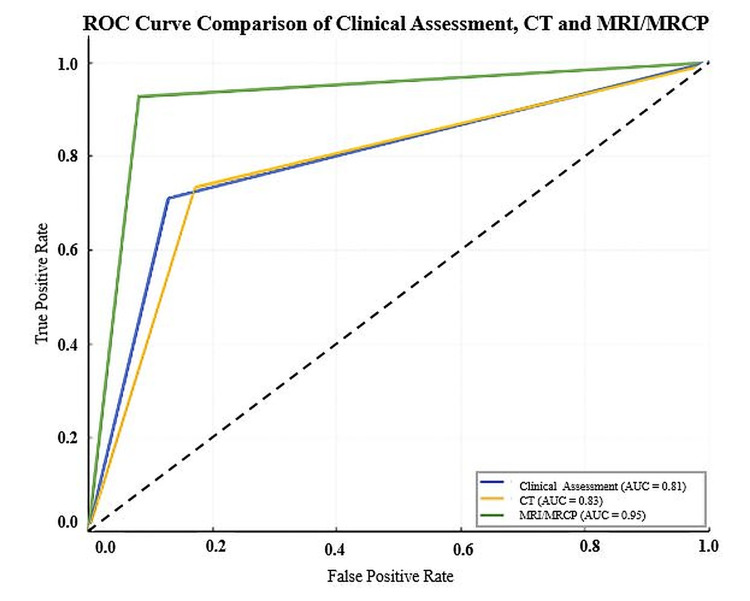
ROC curves comparing the diagnostic performance of MRI/MRCP, CT, and clinical assessment for the detection of common bile duct stones True positive rate: sensitivity; false positive rate: 1 - specificity AUC: area under the curve; ROC: receiver operating characteristic; CT: computed tomography; MRI/MRCP: magnetic resonance imaging with magnetic resonance cholangiopancreatography

ROC curve analysis (Figure 4) further highlighted the superior diagnostic accuracy of MRI/MRCP, with an AUC of 0.95 (95% CI: 0.92-0.98). This was statistically significantly higher than both CT (AUC=0.83; 95% CI: 0.79-0.87; p<0.001) and clinical assessment (AUC=0.81; 95% CI: 0.77-0.85; p<0.001). There was no statistically significant difference in diagnostic performance between CT and clinical assessment (p=0.45). These comparative statistical analyses are presented in Table 3.

**Table 3:** Comparative statistical analysis and ROC curve results AUC: area under the curve; ROC: receiver operating characteristic; CT: computed tomography; MRI/MRCP: magnetic resonance imaging with magnetic resonance cholangiopancreatography

Metric group	Details/comparison	Value (p-value or AUC (95% CI))
Sensitivity	MRI/MRCP vs. CT	<0.001
	MRI/MRCP vs. clinical assessment	<0.001
	CT vs. clinical assessment	0.58
Specificity	MRI/MRCP vs. CT	0.005
	MRI/MRCP vs. clinical assessment	0.04
	Clinical assessment vs. CT	0.04
ROC analysis	MRI/MRCP	0.95 (0.92-0.98)
	CT	0.83 (0.79-0.87)
	Clinical assessment	0.81 (0.77-0.85)
AUC comparison	MRI/MRCP vs. CT	<0.001
	MRI/MRCP vs. clinical assessment	<0.001
	CT vs. clinical assessment	0.45

## Discussion

The principal strength of this study is its direct, head-to-head comparison of three common diagnostic strategies, that is, clinical assessment, CT, and MRI/MRCP, within a large, real-world patient cohort evaluated against a definitive procedural reference standard. The principal finding of this study is that MRI/MRCP demonstrates substantially superior diagnostic accuracy for the detection of CBD stones compared to both multidetector CT and clinical assessment based on ASGE high-risk criteria. With an AUC of 0.95, MRI/MRCP proved to be an outstanding non-invasive diagnostic tool, a finding that aligns with and reinforces previous literature [8,9].

In our cohort, MRI/MRCP yielded a sensitivity of 92.7% and a specificity of 91.9%. This high performance, particularly the excellent sensitivity, is crucial for its role as a "gatekeeper" test to avoid unnecessary invasive procedures like ERCP. A negative MRCP result, given its high NPV (93.4%) in our study, can confidently rule out the presence of CBD stones, thereby sparing patients the risks associated with diagnostic ERCP, such as pancreatitis, hemorrhage, and perforation [3]. Our findings are consistent with prior studies, such as Kondo et al. [3], who reported a sensitivity of 88% for MRCP, although our slightly higher value may reflect advances in MRI technology, including higher field-strength magnets and more sophisticated sequences over the intervening years [9].

The diagnostic performance of CT in our study was modest, with a sensitivity of 73.2% and an accuracy of 78.3%. While CT is invaluable in the acute setting for evaluating undifferentiated abdominal pain, our data confirm its limitations in the specific context of choledocholithiasis. The lower sensitivity is likely due to the difficulty in visualizing small, non-calcified, or isodense stones, which may not be conspicuous against surrounding bile or soft tissue [6,7]. Yeh et al. noted that even with modern techniques, the sensitivity of CT for choledocholithiasis is reported to be between 72% and 88%, which is consistent with our findings [7]. This underscores that while a positive CT scan is useful, a negative scan does not reliably exclude CBD stones, especially in patients with a high clinical suspicion.

Clinical assessment, based on ASGE high-risk criteria, demonstrated a relatively low sensitivity (71.1%) but high specificity (87.1%). This profile highlights its role as an effective triage tool rather than a definitive diagnostic test. High specificity indicates that when high-risk criteria are met (e.g., cholangitis or total bilirubin >4 mg/dL), the likelihood of CBD stones is very high, justifying a direct move to therapeutic ERCP. However, its low sensitivity implies that a significant number of patients with CBD stones do not meet these strict criteria and fall into the intermediate-risk group, for whom further diagnostic imaging is essential. Our study validates that for this large intermediate-risk group, MRI/MRCP is the most accurate non-invasive modality to clarify the diagnosis.

The clinical implications of our findings are clear. In patients with suspected CBD stones who do not meet high-risk criteria for direct-to-ERCP management, MRI/MRCP should be the preferred diagnostic test. It offers the highest accuracy and can prevent the majority of negative diagnostic ERCPs. While CT remains a cornerstone of emergency abdominal imaging, clinicians must be aware of its limited sensitivity for CBD stones and should not hesitate to proceed with MRI/MRCP if clinical suspicion persists despite a negative CT result.

Our study has several limitations. First, its retrospective nature may have introduced selection bias and relied on the quality of existing clinical documentation. Second, the study's confinement to a single city's health system (Baghdad) means the findings may not be fully generalizable to different patient populations or healthcare systems. A significant limitation is that not all patients underwent all three diagnostic evaluations, which precludes a perfectly paired comparison across the entire cohort. Statistical comparisons were performed on available subsets, but the validity is limited by this design, and the specific sample sizes for each paired analysis are not reported. Furthermore, we did not calculate interobserver agreement for the radiological reviews, which leaves the degree of interpretive consistency unquantified. While the study reaffirms the high accuracy of MRI/MRCP, a key limitation is the absence of endoscopic ultrasound (EUS) as a comparator. EUS is a highly accurate modality, potentially superior for detecting small (<5 mm) stones, and its omission limits the direct applicability of our findings for guiding the choice between non-invasive imaging modalities [9,10]. Finally, while radiologists were blinded, the clinicians performing the initial assessment were not, introducing a potential source of bias.

## Conclusions

In the clinical evaluation of suspected CBD stones, where an accurate diagnosis is paramount for guiding patient management, this retrospective study provides a clear and direct comparison of common non-invasive diagnostic strategies. Our findings indicate that MRI/MRCP is a substantially superior modality, demonstrating significantly higher sensitivity, specificity, and overall accuracy compared to both multidetector CT and clinical assessment. This positions MRI/MRCP as a crucial gatekeeper test, particularly for the large cohort of patients classified with an intermediate probability of disease. The high NPV of MRI/MRCP allows clinicians to confidently rule out stones, thereby preventing a majority of patients from undergoing unnecessary, costly, and potentially high-risk invasive procedures like ERCP. While clinical assessment remains a vital first step for triage and CT is a cornerstone of emergency imaging, their limitations in this specific diagnostic context must be recognized. Within the limitations of a retrospective design, our analysis strongly advocates for the integration of MRI/MRCP as the preferred non-invasive imaging investigation to confirm or exclude choledocholithiasis when the diagnosis remains uncertain, ensuring that patient care is both effective and safe.
